# Clinical evaluation of constant rate infusion of alfaxalone–medetomidine combined with sevoflurane anesthesia in Thoroughbred racehorses undergoing arthroscopic surgery

**DOI:** 10.1186/s13028-018-0406-4

**Published:** 2018-09-04

**Authors:** Hirotaka Tokushige, Asuka Kushiro, Atsushi Okano, Tatsuya Maeda, Hideki Ito, Ai Wakuno, Shun-ichi Nagata, Minoru Ohta

**Affiliations:** 1 Racehorse Clinic, Miho Training Center, Japan Racing Association (JRA), 2500-2 Mikoma, Miho-mura, Inashiki-gun, Ibaraki 300-0493 Japan; 2 Racehorse Clinic, Ritto Training Center, JRA, 1028 Misono, Ritto-shi, Shiga 520-3085 Japan; 3 Analytical Chemistry Section, Laboratory of Racing Chemistry, 1731-2 Tokami-cyo, Utsunomiya-shi, Tochigi 320-0851 Japan; 4 Equine Research Institute, JRA, 1400-4 Shiba, Shimotsuke-shi, Tochigi 329-0412 Japan

**Keywords:** Alfaxalone–medetomidine, Constant rate infusion, Equine anesthesia, Racehorse, Recovery, Sevoflurane

## Abstract

**Background:**

Alfaxalone has a number of pharmacological properties which are desirable for constant rate infusion (CRI). Previously, the co-administration of alfaxalone and medetomidine is shown to be suitable for short-term anesthesia in horses. However, the use of alfaxalone–medetomidine CRI with inhalational anesthesia under surgical procedures have not been investigated in clinical cases. The aim of the present study was to evaluate the clinical efficacy of alfaxalone–medetomidine CRI in sevoflurane-anesthetized Thoroughbred racehorses undergoing arthroscopic surgery. Sevoflurane requirement, cardiovascular function, and induction/recovery quality were compared between horses maintained with sevoflurane in combination with medetomidine CRI (3 µg/kg/h) (Group M; n = 25) and those maintained with sevoflurane in combination with alfaxalone–medetomidine CRI (0.5 mg/kg/h and 3 µg/kg/h, respectively) (Group AM; n = 25).

**Results:**

The mean end-tidal sevoflurane concentrations were significantly lower in Group AM (1.8 ± 0.2%) than in Group M (2.4 ± 0.1%). The mean dobutamine infusion rate required for maintaining mean arterial blood pressure within the target values (60–80 mmHg) was significantly lower in Group AM (0.53 ± 0.20 µg/kg/min) than in Group M (0.85 ± 0.32 µg/kg/min). Induction and recovery scores were not significantly different between two groups. However, excitatory response during recovery were observed in five horses in Group AM. The mean plasma alfaxalone concentrations were stable throughout the maintenance period (0.77 ± 0.12 to 0.85 ± 0.13 µg/mL), and decreased significantly immediately after standing (0.32 ± 0.07 µg/mL).

**Conclusions:**

Alfaxalone–medetomidine CRI reduced sevoflurane requirement by approximately 26% with good maintenance of cardiopulmonary function in Thoroughbred racehorses undergoing arthroscopic surgery. Sevoflurane in combination with alfaxalone–medetomidine CRI may be a clinically effective anesthetic technique for Thoroughbred racehorses. However, 20% of horses administered alfaxalone showed remarkable excitatory response during recovery. Greater attention to excitatory response may be advisable if alfaxalone is used for induction or maintenance of anesthesia. A larger study is needed to explore the clinical relevance of these findings.

## Background

Among the volatile anesthetic agents commonly used in equine anesthesia, sevoflurane has the advantages of rapid induction of anesthesia, easy control of anesthetic depth and rapid recovery due to its low blood solubility [1–3]. However, sevoflurane is known to cause dose-dependent cardiopulmonary depression, which increases the risk of peri-anesthetic mortality and death [4–7]. Therefore, balanced anesthesia is often used to reduce the requirements of inhalation anesthetics and to thereby minimize their cardiovascular depressant effects in equine practice [8].

Most balanced anesthetic protocols include the use of an α2-adrenoceptor agonist because of their potent sedative and analgesic effects [8]. One such α2-adrenoceptor agonist is medetomidine, whose short half-life, selectivity, and potency make it suitable for use as a constant rate infusion (CRI) for balanced anesthesia in horses [9–12]. In a previous study, we found that medetomidine CRI (3.0 µg/kg/h) reduced the sevoflurane requirement for arthroscopic surgery by approximately 10% in Thoroughbred racehorses, resulting in good maintenance of cardiopulmonary function, and an improvement in the quality of recovery from anesthesia [13]. However, the anesthetic sparing effect of medetomidine CRI on sevoflurane was insufficient to minimize the negative cardiovascular effects of sevoflurane, and cardiovascular depression during the maintenance period still remained a concern.

Alfaxalone is a synthetic neuroactive steroid that acts on the gamma aminobutyric acid (GABA)_A_ receptors in the central nervous system and produces unconsciousness and muscle relaxation. Several experimental trials of alfaxalone in horses have been reported [14–18]. It is reported that the effects of alfaxalone in relation to induction of anesthesia, recovery quality, and cardiopulmonary response in Thoroughbred horses are similar to the effects of ketamine and thiopental [18], and that alfaxalone has a number of pharmacological properties which are desirable for CRI in horses [14, 19]. It is also reported that the co-administration of alfaxalone and medetomidine as CRI after induction of anesthesia with alfaxalone is suitable for short-term anesthesia in horses undergoing field castration [20]. Therefore, a further reduction in sevoflurane requirement would be expected if alfaxalone–medetomidine CRI was combined with sevoflurane anesthesia.

The purpose of this study was to assess the clinical efficacy of alfaxalone–medetomidine CRI in sevoflurane-anesthetized Thoroughbred racehorses undergoing arthroscopic surgery. In this study, sevoflurane requirement, cardiopulmonary function, and induction/recovery quality were compared between horses maintained with sevoflurane in combination with medetomidine CRI (Group M) and those maintained with sevoflurane in combination with alfaxalone–medetomidine CRI (Group AM).

## Methods

### Animals

Our study included 50 Thoroughbred racehorses with chip fractures of the carpal bones undergoing arthroscopic surgery. The surgery was performed on a single leg or both legs by randomly assigned experienced surgeons. The horses were assigned randomly to Group M or Group AM (25 horses in each group). In Group M, anesthesia was maintained with sevoflurane in combination with medetomidine CRI; in Group AM, anesthesia was maintained with sevoflurane in combination with alfaxalone plus medetomidine CRI. The mean ± standard deviation (SD) age and body weight were, respectively, 3.3 ± 0.5 years old and 470 ± 21 kg in Group M and 3.1 ± 0.7 years old and 465 ± 28 kg in Group AM. All horses were subjected to preanesthetic blood testing and electrocardiography. Food, but not water, was withheld for 12 h prior to anesthesia.

### Experimental protocol

All horses were premedicated with medetomidine (Dorbene; Vetcare Oy, Salo, Finland) (5.0 µg/kg, IV) and midazolam (Dormicum; Astellas Pharma Inc., Tokyo, Japan) (20 µg/kg, IV) together. Anesthesia was induced in the horses in Group M by a rapid injection of 5% guaifenesin (5% Guaifenesin; Shinyo Pure Chemicals Co., Ltd., Osaka, Japan) (100 mg/kg, IV) with thiopental sodium (Ravonal; Mitsubishi Tanabe Pharma Co., Osaka, Japan) (4.0 mg/kg, IV); anesthesia in Group AM was induced by guaifenesin (the same volume as in Group M) with alfaxalone (Alfaxan; Jurox Pty Ltd., Rutherford, Australia) (1.0 mg/kg, IV). A scoring scale of 1–5 (G1: poor, G2: marginal, G3: fair, G4: good, G5: excellent) was used for the subjective assessment of induction of anesthesia [21]. After induction of anesthesia, horses were intubated endotracheally and positioned in dorsal recumbency. Anesthesia was maintained with sevoflurane (Sevofrane; Maruishi Pharmaceutical Co., Ltd., Osaka, Japan) and oxygen (approximately 5 L/min) using an intermittent positive pressure ventilator (MOK 94; Silver Medical Co., Tokyo, Japan) with a peak airway pressure of 25 cmH_2_O to maintain the arterial carbon dioxide tension (PaCO_2_) between 45 and 55 mmHg.

A base-apex lead electrocardiogram was used to monitor heart rate (HR) and rhythm. A 20-G catheter was placed in the facial artery for arterial blood pressure measurements and arterial blood sample collections. Arterial blood pressures were measured directly through the catheter by a transducer system. Respiratory gas was collected continuously, and the end-tidal sevoflurane concentrations (ET_SEVO_) was determined by infrared absorption. The ET_SEVO_ was recorded throughout anesthesia, and HR, systolic arterial blood pressure (SAP), diastolic arterial blood pressure (DAP) and mean arterial blood pressure (MAP) were recorded every 5 min by an anesthesia monitoring system (BP608; Omron Colin Co., Ltd., Tokyo, Japan). Arterial blood samples were collected every 15 min and PaCO_2_, arterial oxygen partial pressure (PaO_2_) and pH were immediately analyzed by a blood-gas analyzer (ABL800 FLEX; Radiometer Co., Ltd., Tokyo, Japan).

Throughout the maintenance period, horses in Group M received medetomidine CRI at a constant rate of 3.0 µg/kg/h and horses in Group AM received medetomidine CRI at a constant rate of 3.0 µg/kg/h and alfaxalone CRI at a constant rate of 0.5 mg/kg/h. The vaporizer setting of sevoflurane was based on observation of standard clinical signs to achieve a surgical plane of anesthesia. Anesthetic depth was judged to be light if movement, brisk palpebral response, spontaneous nystagmus, or sudden changes in arterial blood pressure and HR were observed. Lactated Ringer’s solution was administered intravenously at a rate of approximately 10 mL/kg/h throughout anesthesia. Dobutamine (Dobutrex; Shionogi & Co., Ltd., Osaka, Japan) was infused to maintain MAP between 60 and 80 mmHg.

Horses in the two groups were allowed to recover without assistance and were given no additional sedatives. Oxygen was supplied until adequate spontaneous respiration appeared, and then the endotracheal tube was removed. The behavior and clinical response course of recovery were scored according to Mama’s report [21] as follows: G5 (excellent, single coordinated effort to stand with minimal to no ataxia), G4 (fair, single attempt to stand with some ataxia), G3 (good, quiet recovery with more than one attempt to stand), G2 (marginal, uncoordinated attempts to stand with or without minor injury) and G1 (poor, multiple, uncoordinated attempts resulting in major or life-threatening injury). The number of attempts to stand and the times from the end of anesthesia to the appearance of spontaneous respiration, extubation, first movement, sternal recumbency, first attempt to stand, and standing were recorded.

### Plasma alfaxalone analysis

Blood samples were collected from 10 out of 25 horses in Group AM after induction of anesthesia; at 15, 30, and 45 min after connection to the breathing circuit; and immediately after standing. All blood samples were immediately placed on ice, and then the plasma was separated from the blood and frozen at − 20 °C. Alfaxalone in plasma was extracted by liquid–liquid extraction using methyl tert-butyl ether. The extracted substance was analyzed using liquid chromatography–tandem mass spectrometry consisted of Shimadzu prominence HPLC system (Shimadzu Co., Tokyo, Japan) and AB Sciex QTRAP 4000 mass spectrometer (AB Sciex, Framingham, MA, USA).

### Statistical analysis

Age, body weight, mean ET_SEVO_, mean dobutamine infusion rate, blood gas data, duration of anesthesia, recovery time, induction score, and recovery score were compared between the two groups by using the Mann–Whitney’s U-test. Two-way repeated-measures ANOVA tests were applied to compare cardiovascular data between the two groups. One-way repeated-measures ANOVA tests were applied to compare plasma alfaxalone concentrations in Group AM. The Tukey’s Kramer-test for multiple comparisons was applied when significant differences were identified. Values are given as mean ± SD, and statistical significance was set at *P* < 0.05.

## Results

**Fig. 1:**
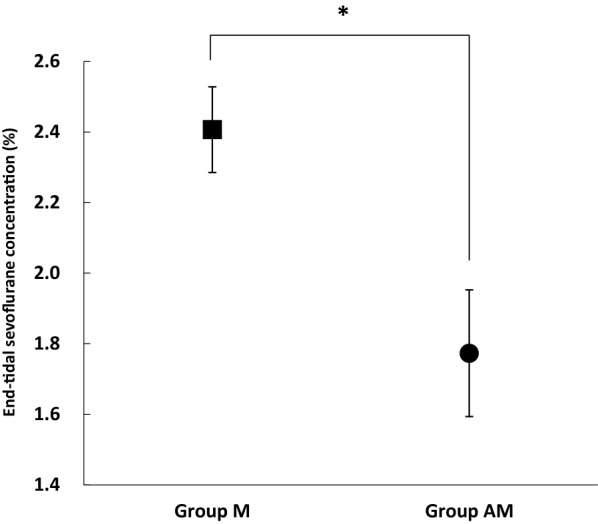
Mean end-tidal sevoflurane concentration during the maintenance of anesthesia. Horses in Group M (n = 25) (black square) were maintained with sevoflurane in combination with medetomidine CRI; horses in Group AM (n = 25) (black circle) were maintained with sevoflurane in combination with alfaxalone–medetomidine CRI. *Significant difference between the two groups (*P *< 0.001), *P*: probability

No abnormality was found in any of the horses based on the results of the preanesthetic blood examination and electrocardiography. There was no significant difference in age or in body weight between the two groups. Duration of the anesthesia was 55 ± 12 min in Group M and 60 ± 11 min in Group AM, and these were not significantly different. The mean ET_SEVO_ values during the maintenance period (Fig. 1) were significantly lower (*P *< 0.001) in Group AM (1.8 ± 0.2%) than in Group M (2.4 ± 0.1%). The cardiovascular parameters HR, SAP, DAP, and MAP did not change significantly throughout the maintenance, and there were no significant differences between the two groups (Table 1). The mean dobutamine infusion rate required to maintain mean arterial blood pressure within the target values (60–80 mmHg) was significantly lower (*P *< 0.001) in Group AM (0.53 ± 0.20 µg/kg/min) than in Group M (0.85 ± 0.32 µg/kg/min). The mean values of PaCO_2_ and PaO_2_ in Group AM (48 ± 3 mmHg and 461 ± 49 mmHg, respectively) were not significantly different from those in Group M (51 ± 3 mmHg and 474 ± 46 mmHg, respectively).

**Table 1:** Cardiovascular parameters during the maintenance of anesthesia in the horses of Group M and Group AM

Variable	Group	Time after connection to breathing circuit (min)				
		0	15	30	45	60
HR (beats/min)	M	30 ± 3	27 ± 3	26 ± 3	27 ± 3	27 ± 3
	AM	31 ± 4	28 ± 4	27 ± 3	27 ± 3	28 ± 3
SAP (mmHg)	M	n/a	93 ± 8	97 ± 8	101 ± 9	104 ± 9
	AM	n/a	94 ± 6	102 ± 10	104 ± 9	101 ± 4
DAP (mmHg)	M	n/a	51 ± 8	54 ± 7	55 ± 7	56 ± 7
	AM	n/a	51 ± 7	59 ± 6	60 ± 7	57 ± 6
MAP (mmHg)	M	n/a	63 ± 8	68 ± 7	70 ± 7	71 ± 7
	AM	n/a	64 ± 7	73 ± 5	74 ± 4	72 ± 5

Data are presented as mean ± standard deviation (SD)

Horses in Group M (n = 25) were maintained with sevoflurane in combination with medetomidine CRI; horses in Group AM (n = 25) were maintained with sevoflurane in combination with alfaxalone–medetomidine CRI

*HR* heart rate, *SAP* systolic arterial blood pressure, *DAP* diastolic arterial blood pressure, *MAP* mean arterial blood pressure, *n/a* not applicable

**Fig. 2:**
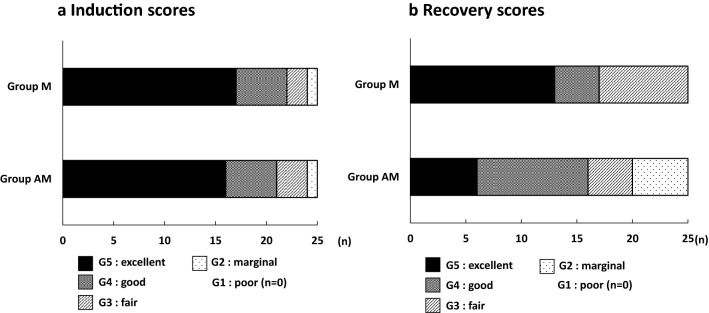
Induction scores (**a**) and recovery scores (**b**). Horses in Group M (n = 25) were maintained with sevoflurane in combination with medetomidine CRI; horses in Group AM (n = 25) were maintained with sevoflurane in combination with alfaxalone–medetomidine CRI

Induction and recovery scores were not significantly different between the two groups (Fig. 2). The scores for recovery in Group AM were G5 for 6 horses, G4 for 10 horses, G3 for 4 horses and G2 for 5 horses. Remarkable excitatory response at attempts to stand were observed in 5 horses classified as G2. The mean times from the end of anesthesia until the appearance of spontaneous respiration, extubation, first movement, sternal recumbency, first attempt to stand, and standing in Group AM (9 ± 5 min, 17 ± 6 min, 44 ± 12 min, 62 ± 14 min, 68 ± 13 min, and 72 ± 14 min, respectively) were not significantly different from those in Group M (10 ± 8 min, 18 ± 8 min, 46 ± 12 min, 61 ± 10 min, 64 ± 10 min and 65 ± 9 min, respectively).

**Fig. 3:**
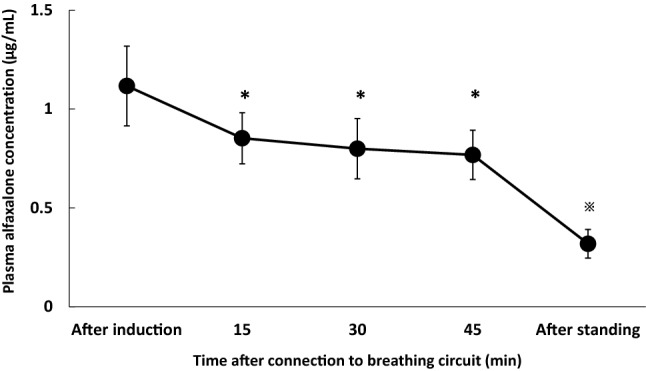
Plasma alfaxalone concentration immediately after induction, during maintenance, and after standing in Group AM. Horses in Group AM (n = 25) were maintained with sevoflurane in combination with alfaxalone–medetomidine CRI. *Significantly different from the values immediately after induction of anesthesia (*P *< 0.01), ^※^significantly different from the values immediately after induction of anesthesia and during sevoflurane maintenance (*P *< 0.01), *P*: probability

The plasma alfaxalone concentrations immediately after induction of anesthesia, during the maintenance of anesthesia, and after standing in Group AM are shown in Fig. 3. The mean plasma alfaxalone concentration immediately after induction (1.12 ± 0.20 µg/mL) was significantly higher than the concentrations during the maintenance and after standing (*P *< 0.01, *P *< 0.01, respectively). The mean plasma alfaxalone concentrations during the maintenance (0.77 ± 0.12 to 0.85 ± 0.13 µg/mL) were stable and did not change significantly throughout the maintenance. The mean plasma alfaxalone concentration significantly decreased immediately after standing (0.32 ± 0.07 µg/mL) (*P *< 0.01).

## Discussion

The ET_SEVO_ providing a surgical plane of anesthesia for horses anesthetized with sevoflurane is reported to be 2.5–2.8% for orthopedic surgery [22]. We have reported that medetomidine CRI reduced the mean ET_SEVO_ concentrations during surgery for arthroscopic surgery by approximately 10% [13]. In the present study, the mean ET_SEVO_ in Group AM necessary to maintain the surgical plane of anesthesia during arthroscopic surgery was 1.8%, which was equivalent to approximately 80% of the minimum alveolar concentration (MAC) of sevoflurane in horses (2.31 ± 0.11%) [23]. The balanced anesthesia used in Group AM resulted in an approximately 26% reduction in ET_SEVO_ compared to that in Group M. All horses used in this study had the same fracture characteristics, and so the surgical stimuli were considered to be of a similar extent in both groups. Therefore, the potent anesthetic effect of alfaxalone is suggested to have compensated for the reduction in sevoflurane concentration.

There is currently limited information regarding the effects that alfaxalone CRI has on the doses of inhalation anesthetics required. In sheep, infusion of alfaxalone at 0.42 mg/kg/h resulted in a 22% reduction in the desflurane expiratory fraction required for maintenance of anesthesia [24]. In the current study, CRI of alfaxalone (0.5 mg/kg/h) resulted in mean ET_SEVO_ values of 1.8% over the duration of the surgical procedure. However, crucially, the design of the present study had limitations with respect to detecting the sevoflurane-sparing effects of alfaxalone CRI because the induction agent used in each group was different (thiopental versus alfaxalone). Nevertheless, Wakuno et al. [18] reported that thiopental and alfaxalone showed similar effects in relation to induction of anesthesia and cardiopulmonary responses. In this study, in anticipation of the clinical applicability of alfaxalone, anesthesia in Group AM was induced by a loading dose of alfaxalone followed by CRI at a dose sufficient to maintain a stable plasma concentration of alfaxalone during the surgical procedure. Although it was difficult to make accurate comparisons owing to the different induction agents and the absence of an experimental MAC reduction study, we considered that alfaxalone CRI had a moderate degree of sevoflurane-sparing effect in horses.

In the present study, the mean ET_SEVO_ values in Group AM were 1.8%; the infusion rates of alfaxalone and medetomidine used were selected on the basis of preliminary studies and experimental work involving these drugs determined in the references of previous reports [13, 24, 25]. Granados et al. [24] reported that a loading dose of alfaxalone of 1.0 mg/kg followed by a maintenance dose of 0.42 mg/kg/h reduced the desflurane requirement in sheep by 22%. Ambros et al. [25] reported that a maintenance dose of 0.42 mg/kg/h produced clinically acceptable anesthetic quality and hemodynamic values ideal for use as a CRI in dogs. When medetomidine CRI (3.0 µg/kg/h) was applied during sevoflurane anesthesia, the sevoflurane dose required for maintenance of anesthesia was significantly lower than when sevoflurane was used alone [13]. In this study, a loading alfaxalone dose of 1.0 mg/kg followed by a maintenance dose of 0.5 mg/kg/h was considered to contribute to the ET_SEVO_ value of 1.8% of in Group AM. In addition, the infusion rate of alfaxalone effectively maintained stable plasma alfaxalone concentrations and there were no observations of light anesthesia with surgical stimulation. Although meaningful comparisons between previous studies and the present study are difficult, the infusion of alfaxalone and medetomidine would be expected to provide an effective balanced anesthesia in horses similar to that in other species because of the adequate anesthetic depth throughout the maintenance period.

It is essential that any anesthetic technique used in horses maintains adequate cardiopulmonary function. In our study, there was no significant difference between Group M and Group AM in terms of heart rate and arterial blood pressure. HR was stable and was maintained at an adequate value during anesthesia. MAP in Group AM was maintained within the target values (60–80 mmHg) throughout the operation. Sevoflurane induces a dose-dependent decrease in hemodynamic variables in horses, so reducing the dose of sevoflurane may promote cardiovascular stability and improve perianesthetic care [1, 6, 26]. Hence, the reduction in dobutamine requirement is suggested to be a result of the better cardiovascular function. On the other hand, the difference in the induction agents used might also have influenced the dobutamine infusion rates in the present study. There is limited information regarding the cardiopulmonary effects of alfaxalone in horses, but in dogs, a 2-h infusion of alfaxalone resulted in only mild hemodynamic changes, with cardiac variables (cardiac index, heart rate, systemic vascular resistance, arterial blood pressure) remaining stable and not differing significantly from baseline values [25].A previous study in horses showed no significant difference between thiopental and alfaxalone in terms of their effects on arterial blood pressure [18]. From those results it is suggested that the combination alfaxalone–medetomidine CRI minimized cardiovascular depression as a result of reducing the sevoflurane requirement, and that alfaxalone–medetomidine CRI contributed to the significant decrease in the requirement of dobutamine for maintenance of MAP within the target values. However, the level of improvement of cardiovascular depression was moderate. Therefore, we consider that small to moderate amounts of inotropic agents will still be necessary.

With respect to effects in the induction phase, there was no significant difference in induction score between the two groups. The induction dose of alfaxalone used in the current study was chosen based on the results of a previous study that reported there was no statistically significant difference in the quality of anesthetic induction between alfaxalone (1.0 mg/kg, IV) and thiopental (4.0 mg/kg, IV), and that all inductions were of an acceptable quality [18]. Therefore, no difference in induction grade between alfaxalone and thiopental was recognized in the present study.

In the recovery phase, the qualities were clinically acceptable in Group AM; however, remarkable excitatory response at attempts to stand were observed in 5 out of 25 horses. Recovery quality depends on many factors including the horse’s physical status, duration of anesthesia, and the anesthetic drugs used [27, 28]. It was considered that sevoflurane would have been washed out from the body more quickly in Group AM than in Group M because the mean ET_SEVO_ values in Group AM were significantly lower than those in Group M. However, there was no significant difference in recovery quality between the two groups. A major concern when using alfaxalone infusion to maintain anesthesia is that accumulation of the drug or its active metabolites may occur, resulting in prolonged and potentially ataxic and uncontrolled recoveries [20]. Goodwin et al. [29] reported unacceptable recoveries in horses recovering from 3-h general anesthesia with an infusion of alfaxalone of 3 mg/kg/h. Others reported that alfaxalone induced central nervous system excitation and that induction of anesthesia with alfaxalone was associated with poor recovery [30, 31]. In the current study, the adverse effects of alfaxalone might remain in the attempt to stand because the plasma alfaxalone concentration after standing in Group AM was 0.32 ± 0.07 µg/mL. The excitation of 20% of horses during recovery has potential for an increased risk of major or life-threatening injury when using alfaxalone. We consider that horses administered alfaxalone should be given additional sedatives and be assisted, and so recovery quality might be improved. Anyway, the present results indicate that further investigation is required to explore the clinical relevance of excitatory response induced by alfaxalone because 25 horses are not many with regards to more severe consequences.

## Conclusions

Alfaxalone–medetomidine CRI reduced sevoflurane requirement by approximately 26% with good maintenance of cardiopulmonary function in Thoroughbred racehorses undergoing arthroscopic surgery. Sevoflurane in combination with alfaxalone–medetomidine CRI may be a clinically effective anesthetic technique for Thoroughbred racehorses. However, 20% of horses administered alfaxalone showed remarkable excitatory response during recovery. Greater attention to excitatory response may be advisable if alfaxalone is used for induction or maintenance of anesthesia. A larger study is needed to explore the clinical relevance of these findings.

## Abbreviations

ANOVA: analysis of variance
CRI: constant rate infusion
DAP: diastolic arterial blood pressure
ET_SEVO_: end-tidal sevoflurane concentrations
HR: heart rate
IV: intravenous
MAP: mean arterial blood pressure
*p*: probability
PaCO_2_: arterial carbon dioxide tension
PaO_2_: arterial oxygen partial tension
SAP: systolic arterial blood pressure
SD: standard deviation
